# Semiparametric model selection for identification of environmental covariates related to adult groundfish catches and weights

**DOI:** 10.1038/s41598-021-89398-8

**Published:** 2021-05-11

**Authors:** Hannah E. Correia

**Affiliations:** 1 Department of Biostatistics, Harvard University, Boston, MA 02115 USA; 2 Harvard Data Science Initiative, Harvard University, Cambridge, MA 02138 USA

**Keywords:** Biooceanography, Community ecology, Ecological modelling, Marine biology, Marine chemistry, Physical oceanography

## Abstract

Ecologists and fisheries managers are interested in monitoring economically important marine fish species and using this data to inform management strategies. Determining environmental factors that best predict changes in these populations, particularly under rapid climate change, are a priority. I illustrate the application of the least squares-based spline estimation and group LASSO (LSSGLASSO) procedure for selection of coefficient functions in single index varying coefficient models (SIVCMs) on an ecological data set that includes spatiotemporal environmental covariates suspected to play a role in the catches and weights of six groundfish species. Temporal trends in variable selection were apparent, though the selection of variables was largely unrelated to common North Pacific climate indices. These results indicate that the strength of an environmental variable’s effect on a groundfish population may change over time, and not necessarily in-step with known low-frequency patterns of ocean-climate variability commonly attributable to large-scale regime shifts in the North Pacific. My application of the LSSGLASSO procedure for SIVCMs to deep water species using environmental data from various sources illustrates how variable selection with a flexible model structure can produce informative inference for remote and hard-to-reach animal populations.

## Introduction

The northern Pacific system is controlled by multiple interdecadal patterns of climate variability that stem from different physical sources^1^. Groundfish populations in the northeastern Pacific Ocean followed the six- to 12-year warming and cooling periods of the El Niño-Southern Oscillation (ENSO)^2^. Sea surface temperature and pressure changes in the North Pacific are captured by the Pacific Decadal Oscillation (PDO), which is separate from ENSO behavior in the region^3^. A third climate cycle described more recently and termed the Northeast Pacific Gyre Oscillation (NPGO) follows variations in ocean nutrient cycling and phytoplankton abundance and plays a role in the larger system of climate variability with ENSO and PDO^4,5^.

Dramatic, permanent changes in marine species compositions in response to shifts in climate modes, commonly referred to as regime shifts, such as the strong one observed in 1976-1977 in the northern Pacific Ocean may be the convergence of several climate patterns switching phases within the same time period^6,7^. This switching of regimes makes it difficult to identify which specific patterns are culprits in affecting distinct marine populations^8,9^. Many studies on fisheries systems continue to focus on these interdecadal climate modes as primary sources of population variability of marine fishes. However, climate modes alone are insufficient to accurately describe variability found in many commercially valuable marine populations on multi-year scales^10^. Other sources of oceanic variability not captured by climate modes exist and should be considered when attempting to create accurate models to describe and predict changes in marine populations, even in areas that appear to be dominated by shifts in climate regimes. The complicated interplay of ocean-climate systems in the North Pacific region makes it difficult to identify which and how specific indices are culprits in affecting distinct marine populations.

Many of the marine fishes in the northern Pacific Ocean are commercially important species that contribute significantly to the economy of the United States and are important sources of food both domestically and internationally^11–13^. Several of these populations are managed by international or regional fishing commissions to control commercial harvests and monitor population health^11,14–16^. These organizations are becoming more concerned about the role climate plays in maintaining healthy fish populations, especially as marine fishes do not recover from population collapses as quickly as previously believed^17^. Fishing activities are increasingly concentrated on deeper-dwelling species^18,19^. While focus on the effect of various climate modes has dominated ecological research on fishes in the North Pacific region, relationships between the marine environment and atmospheric trends are nuanced and may involve complex lagged effects, particularly for deepwater populations^20^. Organisms that inhabit the deep ocean are also problematic to study, as they are not adapted to surface-level conditions and prove difficult to sample and keep alive, making experiments in laboratory conditions impossible or prohibitively expensive. Determining which specific environmental variables contribute to fluctuations in the populations of these species from observational data would represent major progress in discerning the impact of climate variability on marine ecosystem health and how those changes affect the economy and food security. A model structure able to accommodate a suite of environmental variables that vary spatiotemporally would be necessary to examine effects of many environmental covariates on deepwater marine populations simultaneously.

Consider the single index varying coefficient model (SIVCM) of the form

1 $$\begin{aligned} y_{i} = \{G({\varvec{\theta }}_{0}^{T}Z_{i})\}^{T}X_{i}+\varepsilon _{i}, \qquad i=1,\dots ,n \;, \end{aligned}$$

where $y_{i}$ is the response, $Z=(z_{1i}, z_{2i}, \ldots , z_{ti})^T$ and $X=(x_{0i}, x_{1i}, \ldots , x_{pi})^T$ with $x_{0i} = 1$ are predictor variables, ${\varvec{\theta }}_{0}$ is a vector of unknown coefficients representing the single-index direction, $G(\cdot ) = (g(\cdot )_0, \ldots , g(\cdot )_p)^T$ are nonparametric coefficient functions, and $\varepsilon$ are the random errors^21^. By setting *Z* to longitude, latitude, and time triplets, the SIVCM is a convenient structure for incorporating spatiotemporal effects for multiple environmental predictors in *X*. Spatiotemporal variation among values is incorporated through three-dimensional functions based on spline smoothing^22–27^, accounting for spatial and temporal autocorrelation and improving prediction and inference^28,29^. Similar structures have been widely used in the related spatially-varying coefficient models^30,31^ and temporally-varying coefficient models^32^, including applications in forestry^33^, ecology^34^, and economics^35,36^. Spline-based methods, which are often used to estimate $G(\cdot )$ in SIVCMs^37,38^, are more robust for spatially correlated data and do not require the spatial variation to be specified by a functional form^39,40^.

For such models, selection of important predictor variables in *X* is typically of interest. Forward selection, backwards elimination, and stepwise selection methods are unstable for models with many predictors and even with advancements to the algorithms, these methods are considered sub-optimal for variable selection, particularly for high-dimensional models^41^. Penalty-based regression procedures, such as ridge regression and least absolute shrinkage and selection operator (LASSO) estimation, penalize large regression coefficients to reduce overfitting. LASSO additionally performs variable selection by penalizing small regression coefficients to zero, effectively removing these coefficients from the model^42^. LASSO works particularly well for models with many predictors because it shrinks large coefficients to zero rather than minimizing them, and it is computationally efficient^43^. Group LASSO incorporates information about groupings of variables into the penalty function, which is particularly important for categorical predictor variables^44^. While selection for varying coefficient models (VCMs), a lower-order relative of the SIVCM, have built on both the smoothly clipped absolute deviation (SCAD) and LASSO approaches^45–48^, selection procedures of the single-index direction coefficients or the functions in SIVCMs have primarily used SCAD penalties^49–51^. SCAD procedures are unbiased, but they are sensitive to initial estimation and parameter tuning^48^. LASSO procedures are typically simpler to implement than SCAD, and group LASSO correctly selects important variables for VCMs where the number of dimensions far exceeds the number of observations^52^. In this analysis, I used a combination of least squared-based spline estimation and group LASSO (LSSGLASSO) proposed by Sun et al.^53^ to select coefficient functions and estimate the index parameters in a SIVCM of spatiotemporally-varying environmental covariates potentially contributing to changes in groundfish populations in the North Pacific Ocean. With this application, I aimed to establish relevant environmental factors that influence populations of focal groundfish species in this region.

## Methods

Annual surveys of several groundfish species are taken at established locations in the waters along the coast of Alaska by the Alaska Fisheries Science Center (AFSC), a division of the National Oceanic and Atmospheric Administration (NOAA). Catch per unit effort (CPUE), also referred to as catch rate, and mean weight in kilograms of six groundfish species determined at each location for each survey year were obtained for years 1979–2013^54,55^. The six groundfish species of focus in this analysis were Pacific cod (*Gadus macrocephalus*), Pacific halibut (*Hippoglossus stenolepis*), sablefish (*Anoplopoma fimbria*), rougheye rockfish (*Sebastes aleutianus*), shortraker rockfish (*Sebastes borealis*), and shortspine thornyhead (*Sebastolobus alascanus*). Air temperature in degrees Celsius (ATMP), sea level pressure in hPa (PRES), wind speed in meters per second averaged over eight-minute periods (WSPD), sea surface temperature in degrees Celsius (WTMP), and the average height in meters of the highest one-third of all waves in 20-minute sampling periods (WVHT) measured daily from buoys in the Gulf of Alaska were obtained from the National Data Buoy Center and summarized by monthly means^56^. Temperature in degrees Celsius measured at the sea floor (hereafter bottom temperature) was obtained from the AFSC Resource Assessment and Conservation Engineering (RACE) Division’s bottom trawl surveys^57^. Zooplankton biomass volume given in number per cubic meter (hereafter plankton) were obtained from the NOAA’s Coastal and Oceanic Plankton Ecology, Production, and Observation Database^58^. Alkalinity (Alk), chlorophyll (Chl), nitrate (NO3), dissolved oxygen (Oxy), phosphate (Phos), and silicate (Sil) concentrations at depths of 75, 400, and 900 meters were obtained from the NOAA’s World Ocean Database^59^.

Since environmental data can include hundreds of predictor variables with potential multi-collinearity, a practical method for reducing variables before performing group LASSO is necessary for the LSSGLASSO procedure to be computationally feasible. Therefore, backward variable elimination of explanatory environmental variables using computed variance inflation factors (VIF) was performed. The VIF for explanatory variable *i* was calculated as $1/(1-R_i^2)$ where $R_i^2$ was the correlation coefficient of the linear model with variable *i* regressed against all other explanatory variables. VIFs for all explanatory variables were computed, and the variable with largest VIF that exceeded a value of 5 was removed^60,61^. The process was repeated after each removed variable, until no variables had a $\text {VIF}>5$. None of the environmental variables considered for inclusion in the SIVCM had a $\text {VIF}>5$. The max VIF for any explanatory variables included in the final model was 2.64.

Measurements of environmental variables were not recorded at the exact same locations across all years for which groundfish surveys were performed, thus spatiotemporal interpolation via inverse distance weighting was used to obtain monthly environmental measures for 1979–2013 at the precise locations where MESA surveys were conducted^62,63^. For all environmental variables, a seasonal amplitude was then calculated for each survey year for all locations. For physical variables ATMP, PRES, WSPD, WTMP, WVHT and bottom temperature, seasonal amplitude was defined as the mean of June, July, and August averages minus the mean of December, January, and February averages. This was owing to the fact that temperatures maximize in the summer and minimize in winter and winter storms enable strong mixing of ocean nutrients^64,65^. Seasonal amplitudes for chemical and biological variables including plankton, Chl, Alk, NO3, Oxy, Phos, Sal, and Sil were calculated as the mean of August, September, and October averages minus the mean of March, April, and May averages. Zooplankton biomass increases in May after winter vertical mixing of deepwater nutrients and benefits from spring phytoplankton blooms but has not yet been depleted by grazing from summer-migrating pelagic fishes and cephalopods^66,67^. Therefore nutrients are generally maximal in the spring and minimal in fall after depletion by phytoplankton, whereas zooplankton and chlorophyll would be maximal in fall after nutrient consumption and growth over the summer in the North Pacific region^68–70^.

The AFSC groundfish surveys sample only adults^55^, and groundfish recruitment is related to the effects of physical and biological variables on early life stages^71^. Therefore, all environmental predictors were lagged based on the sexual maturity of the focal groundfish. Sablefish and Pacific cod female maturity is reached around 5 years old^72,73^. Average age of Pacific halibut females that have reached sexual maturity is 12 years, while for males reaching maturity the average age is 8 years^74^. Rockfish sexual maturity is typically attained from 3 to 7 years of age^75^. Therefore, lags of 5 years were used for environmental variables in models with sablefish, rockfish, or Pacific cod CPUEs and weights as responses. Lags of 10 years were used for environmental variables in models with responses of Pacific halibut CPUE and weight. These lags were also supported by the findings of Sun et al.^53^.

A SIVCM of the form given in (1) was fit, where $y_{i}$ was the CPUE or mean weight of a groundfish at each location for each year, $X=(\text{ x}_{0i},\ldots ,\text{ x}_{25i})^{T}$ with $\text{ x}_{0i}=1$ and $\text{ x}_{1i},\ldots ,\text{ x}_{25i}$ were the lagged seasonal amplitudes of environmental variables described previously, and $Z=(z_{1i},z_{2i},z_{3i})^{T}$ were the longitude, latitude, and year for each observation. Selection of important variables was performed per year and over all years using the LSSGLASSO procedure^53^. The choice of knots for the B-spline approximation of the coefficient functions and the optimization of the tuning parameter in the LSSGLASSO procesure are covered in detail in Sun et al.^53^ The optimal tuning parameter was chosen using a BIC-type selection criterion, while the number of knots was fixed at eight. The number of knots can be chosen using a selection criterion such as GCV or BIC, but since basis expansion was only used for selection of the coefficient functions and every function was re-estimated using local linear regression, the choice of the number of knots does not affect the results^53^. The performance of the group LASSO procedure was tested against procedures where the group LASSO penalty was replaced by either a group LASSO with ridge penalty or a group SCAD with ridge penalty, and there were no appreciable differences between the three selection procedures. Heat maps were used to visualize selection of variables for each groundfish species. After selection, the SIVCM for all years of sablefish CPUE was refit with scaled $y, X, \text {and}\; Z$ using only variables selected by LSSGLASSO. The selected coefficient functions for important predictors of sablefish CPUE were plotted to provide a detailed example of interpretation for the SIVCM functions.

To further explore if regional climate conditions influenced the selection of variables each year, I considered potential relationships between the selection of a variable over time and three climate indices that influence environmental systems in the North Pacific region. The Pacific Decadal Oscillation (PDO) monthly index, multivariate El Niño/Southern Oscillation bi-monthly index (MEI), and North Pacific Gyre Oscillation (NPGO) monthly index were obtained for 1979–2013^76–78^. Seasonal amplitude for climate indices was defined for each year as the mean of June, July, and August minus the mean of December, January, and February, since climate indices typically describe physical environmental conditions that contribute to temperature and pressure changes affecting ocean nutrient mixing^79,80^. Logistic regression was then used to fit the model $g({\mathbb {E}}(y_i)) = \beta _0 + \beta _{k} x_{ki}$ for years $i=1985, \ldots , 2013$ for each variable within a groundfish response, where $g(\cdot )$ was a log-link function; $\beta _0$ was the intercept term; $\beta _k$ were linear coefficients, $k \in \{ \text {PDO, MEI, NPGO} \}$; $x_{ki}$ were seasonal amplitudes of index *k* lagged by 5 years when considering the responses of all groundfish except those of Pacific halibut, which were lagged 10 years; and $y_i$ were binary indicators of whether a variable was or was not selected for each year. A Chi-square test was used to compare the models to an intercept-only model. *P*-values from the Chi-square test were adjusted within groundfish response to control for the false discovery rate (FDR) of multiple testing using the Benjamini-Hochberg procedure^81^.

Finally, it was necessary to determine whether PDO, MEI, or NPGO were good predictors of groundfish catches and weights and if the effect of a climate index on the selection of variables was related to that index’s direct relationship to the groundfish populations. I fit generalized additive models (GAMs) of the form

$$\begin{aligned} {\mathbb {E}}(y_i) = f_0 + f_1(\text {PDO}_i-\text {lag}) + f_2(\text {MEI}_i) + f_3(\text {NPGO}_i), \qquad i = 1985, \ldots , 2013 \;, \end{aligned}$$

with lagged seasonal amplitudes of PDO, MEI, and NPGO as three additive smooth predictors and groundfish CPUEs or weights as responses $y_i$^26^. Relationships of climate to groundfish responses cannot be assumed to be linear^82–84^. Therefore, the GAM structure was preferred over a generalized linear model, because it permits nonlinear relationships between predictors and the expected response. *P*-values were estimated for each smooth predictor in each model and plotted in a heatmap for efficient visualization.

## Results

**Figure 1:**
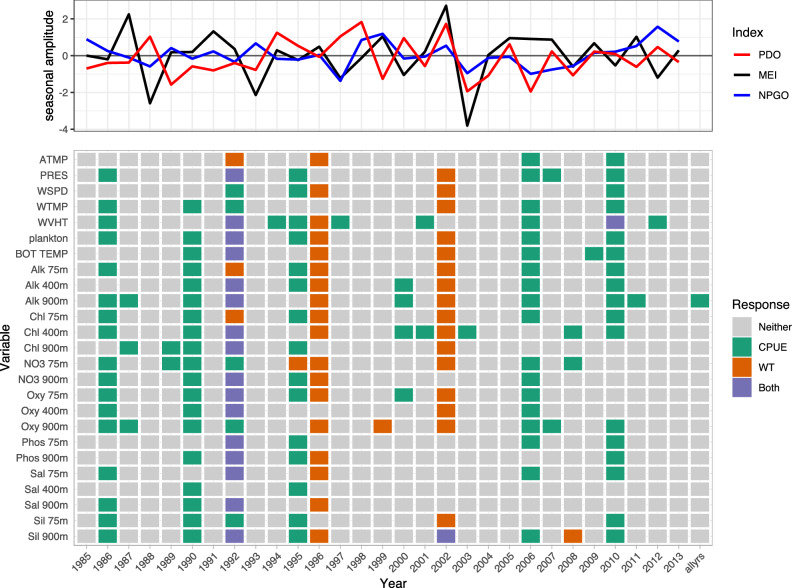
Variable selection analyzed for each available year and all years together for the SIVCMs with Pacific cod CPUE or WT as the response. X-axis represents years for which selection was performed, and all available years of data used in selection labeled as “allyrs”. Y-axis contains all variables in SIVCM from which selection was performed. Colors indicate selection of variable(s) important to CPUE, WT, neither response, or both responses, along with years missing either CPUE or WT response values. Line plot at top shows lagged seasonal amplitude values for the Pacific Decadal Oscillation (PDO), multivariate El Niño/Southern Oscillation (MEI), and North Pacific Gyre Oscillation (NPGO) for corresponding years.

**Figure 2:**
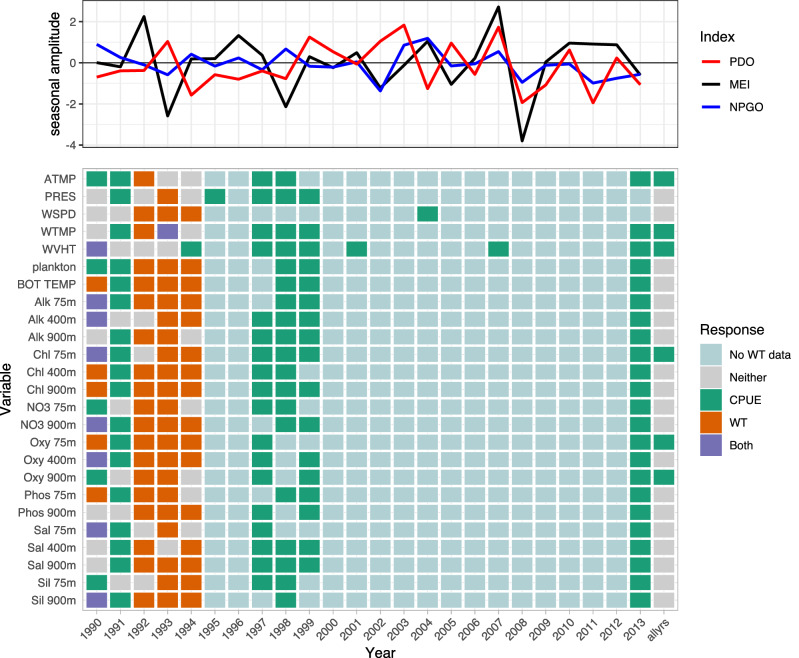
Variable selection analyzed for each available year and all years together for the SIVCMs with Pacific halibut CPUE or WT as the response. Axes, colors, and line plot are as described in Fig. 1.

**Figure 3:**
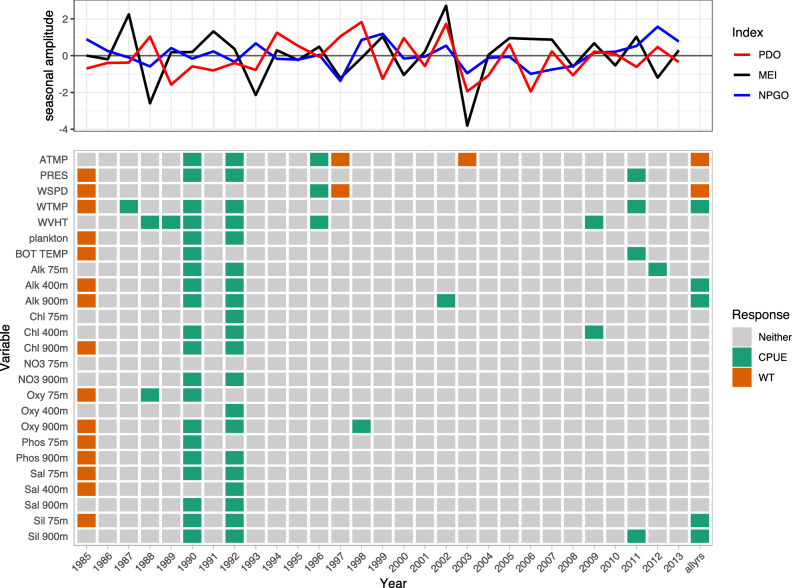
Variable selection analyzed for each available year and all years together for the SIVCMs with sablefish CPUE or WT as the response. Axes, colors, and line plot are as described in Fig. 1.

Variables consistently selected by LSSGLASSO as important to Pacific cod CPUE were WVHT, Alk 900m, Chl 400m, Oxy 900m, Sil 900m, PRES, plankton, Alk 400m, NO3 75m, and Oxy 75m, which usually were in years 1986, 1990, 1992, 1995, 2006, and 2010 (Fig. 1, green). Variables consistently selected in 1992, 1996, and 2002 for Pacific cod weight were Sil 900m, WVHT, plankton, bottom temperature, Alk 75m, Alk 400m, Alk 900m, Chl 75m, Chl 400m, NO3 75m, Oxy 75m, and Oxy 900m (Fig. 1, orange). Shared variables selected for both CPUE and weight of Pacific cod were numerous in 1982 (Fig. 1, purple). WVHT, WTMP, ATMP, plankton, Alk 75m, and NO3 900m in years 1990–1991, 1997–1999, and 2013 were selected variables for Pacific halibut CPUE, whereas variables important for Pacific halibut weight included bottom temperature, Alk 75m, Chl 400m, Chl 900m, NO3 900m, Oxy 75m, Oxy 400m, and Sil 900m for years 1990 and 1992–1994 (Fig. 2). ATMP, WTMP, WVHT, Chl 75m, Oxy 75m, and Oxy 900m were important variables for Pacific halibut CPUE when all years of data were considered together. WTMP, WVHT, Alk 900m, and Sil 900m were most often selected as important for sablefish CPUE, while WSPD and ATMP were the only variables selected as important to sablefish weight more than once over the 29-year period of record and were the only variables important to sablefish weight when all years of data were considered together (Fig. 3).

**Figure 4:**
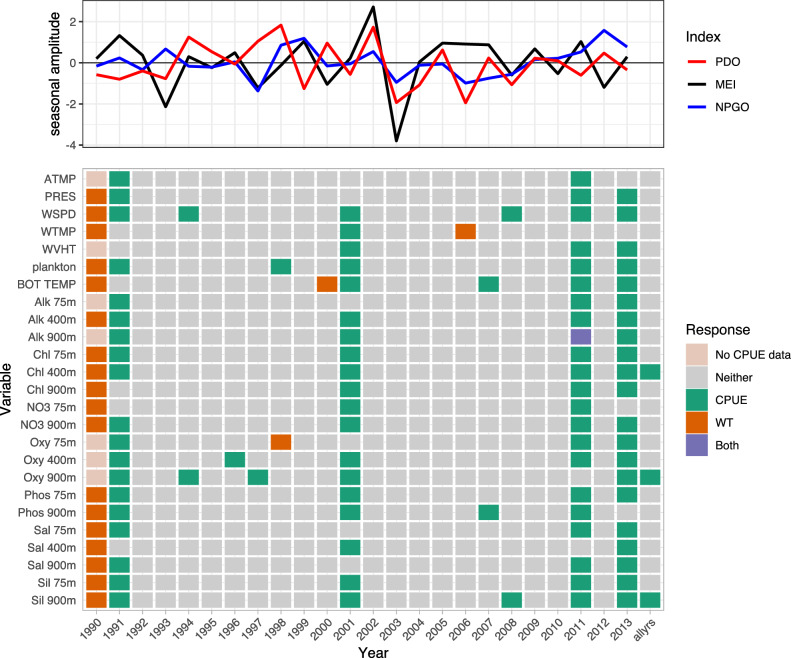
Variable selection analyzed for each available year and all years together for the SIVCMs with rougheye rockfish CPUE or WT as the response. Axes, colors, and line plot are as described in Fig. 1.

**Figure 5:**
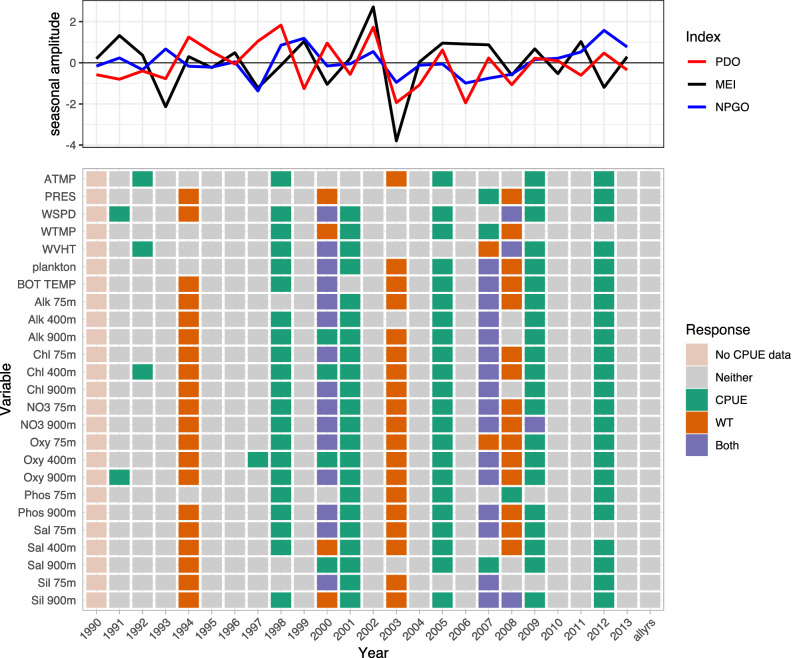
Variable selection analyzed for each available year and all years together for the SIVCMs with shortraker rockfish CPUE or WT as the response. Axes, colors, and line plot are as described in Fig. 1.

**Figure 6:**
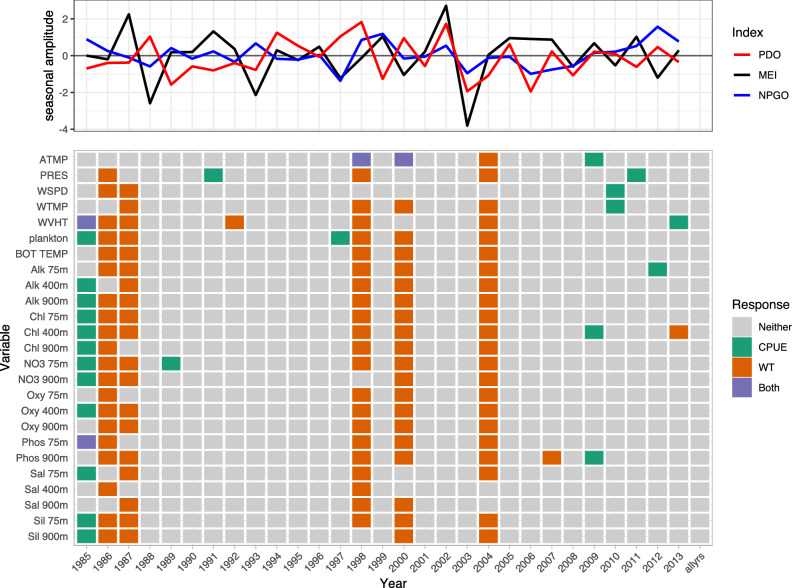
Variable selection analyzed for each available year and all years together for the SIVCMs with shortspine thornyhead CPUE or WT as the response. Axes, colors, and line plot are as described in Fig. 1.

A wide array of environmental variables were selected for rougheye rouckfish CPUE consistently and almost exclusively in years 1991, 2001, 2011, and 2013, whereas 1990 was the only year when more than one variable was selected for rougheye rockfish weight (Fig. 4). The most often selected variables related to rougheye rockfish CPUE were WSPD, Oxy 900m, Sil 900m, plankton, Chl 400m, and Oxy 400m, while Chl 400m, Sil 900m, and Oxy 900m were important when all years were considered together. In the case of shortraker rockfish, most environmental variables were selected as important for both CPUE and weight in 2000 and 2007 (Fig. 5, purple). WSPD, Chl 400m, Oxy 400m, and Oxy 900m were most often selected over time for shortraker rockfish CPUE, while 900m depths of NO3, Sil, Phos, and Oxy, 75m depths of Alk, Chl, NO3, Oxy, and Sal, and bottom temperature were most often selected as important to shortraker rockfish weight over all 29 years (Fig. 5). ATMP, WVHT, and Phos 75m were shared as selected variables for shortspine thornyhead CPUE and weight, and many environmental variables contributed to shortspine thornyhead weight in 1986–1987, 1998, 2000, and 2004 (Fig. 6). ATMP was selected most often for shortspine thornyhead CPUE, while WVHT, Chl 400m, and Phos 900m were consistently selected as important to shortspine thornyhead weight over the 29-year period.

Only Pacific cod CPUE, Pacific halibut CPUE, sablefish CPUE and weight, and rougheye rockfish CPUE had variables selected as important predictors when performing selection on data including all available years (Figs. 1, 2, 3, 4). Alk 900m was a common selected variable for Pacific cod and sablefish CPUEs for all years included in the selection procedure, while WTMP was selected for both sablefish and Pacific halibut CPUEs for all years.

**Figure 7:**
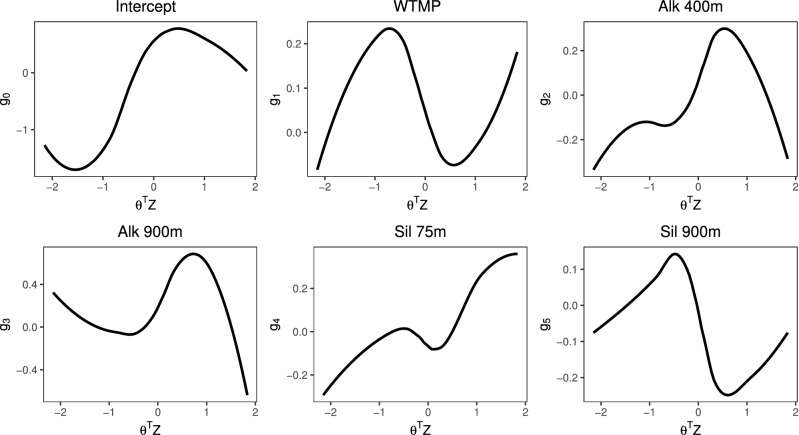
Coefficient functions selected and estimated by LSSGLASSO as important predictors of sablefish CPUE.

I plotted the selected coefficient functions of WTMP, Alk 400m, Alk 900m, Sil 75m, and Sil 900m for sablefish CPUE using all years of data to provide a detailed example of interpretation of the SIVCM coefficient functions (Fig. 7). Parameters representing the single-index direction estimated by the LSSGLASSO procedure were ${\widehat{{\varvec{\theta }}}}^T=(0.9346, -0.3380, -0.1109)$. The relationship between sablefish CPUE and space-time is highly nonlinear, as seen in the intercept function ($g_0$). For a given location and year, we can express the single-index direction as ${\varvec{\theta }}^T Z = 0.9346 z_1 - 0.3380 z_2 - 0.1109 z_3$, where $z_1$ is longitude, $z_2$ is latitude, and $z_3$ is the year. For ${\varvec{\theta }}^T Z$ to increase, either longitude increases (moving eastward) or latitude decreases (moving southward). As time moves forward, i.e. year increases, $\theta ^T Z$ decreases. Fixing two variables in *Z* while moving the third allows us to consider how each important variable in *X* affects the response as ${\varvec{\theta }}^T Z$ changes. For example, for a fixed latitude and year, increasing longitude leads to increasing ${\varvec{\theta }}^T Z$, which was associated with increasing, decreasing, and increasing effect of WTMP on sablefish CPUE ($g_1$ in Fig. 7). So for low and high (east and west) longitude values within the range of the data, increasing WTMP was associated with increasing sablefish CPUE for fixed latitude and year. For medium longitude values, increasing WTMP was accompanied by decreasing sablefish CPUE, ceteris paribus. This same nonlinear relationship was also observed for Sil 900m on sablefish CPUE (Fig. 7, $g_5$). Alternatively for a fixed longitude and year, decreasing latitude (moving southward) precipitates an increase in ${\varvec{\theta }}^T Z$, which was associated with an increasing and then decreasing effect of Alk 400m on sablefish CPUE ($g_2$ in Fig. 7). Hence for southern locations with fixed longitude and year, increasing Alk 400m was associated with decreasing sablefish CPUE. As another option for interpretation, increasing year while fixing longitude and latitude leads to a decrease in ${\varvec{\theta }}^T Z$. Therefore for a fixed longitude-latitude pair, increasing years was associated with increasing, decreasing, then increasing effects of Alk 900m on sablefish CPUE (Fig. 7, $g_3$). Thus in early and late years (e.g. 1985–1988 and 2010–2013) for a fixed location, increasing Alk 900m was related to increasing sablefish CPUE.

**Figure 8:**
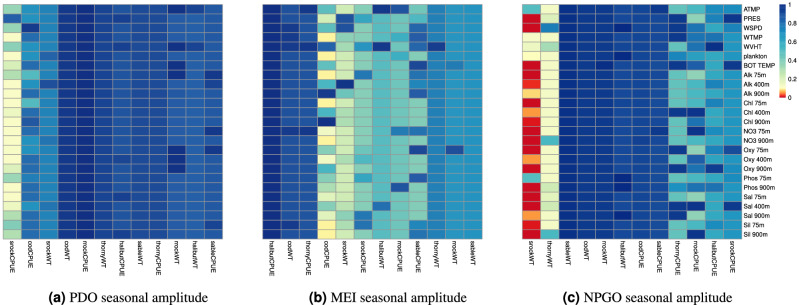
FDR-corrected *P*-values for relationship between selection of each environmental variable contributing to groundfish CPUEs or WTs each year and lagged seasonal amplitudes of climate indices PDO (**a**), MEI (**b**), and NPGO (**c**) fitted using logistic regression. Abbr: cod = Pacific cod; halibut = Pacific halibut; sable = sablefish; rrock = rougheye rockfish; srock = shortraker rockfish; thorny = shortspine thornyhead; CPUE = catch per unit effort; WT = mean weight in kg. *P * < 0.05 are indicated with red.

Lagged seasonal amplitude of NPGO was related to the selection or non-selection of PRES, WSPD, BOT TEMP, Alk 75m, Alk 400m, Chl 75m, Chl 900m, NO3 75m, NO3 900m, Oxy 75m, Oxy 900m, Phos 900m, Sal 75m, Sal 400m, Sil 75m, and Sil 900m for shortraker rockfish weight (Fig. 8). Seasonal amplitudes of PDO, MEI, and NPGO climate indices in the North Pacific were not related to the selection of any environmental variables for any other groundfish responses.

**Figure 9:**
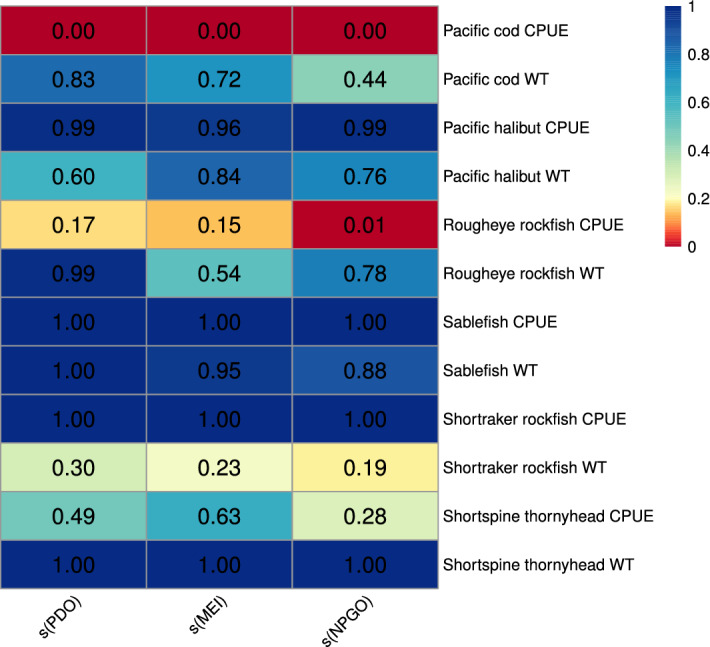
Heatmap of *P*-values for nonlinear relationship between groundfish CPUEs or WTs each year and lagged yearly seasonal amplitudes of climate indices PDO, MEI, and NPGO fitted using generalized additive models (GAMs). Colors and abbr. are as described in Fig. 8.

Lagged seasonal amplitudes of PDO, MEI, and NPGO were significant predictors of Pacific cod CPUE, while PDO and MEI were not significant predictors for any of the other groundfish responses (Fig. 9). Lagged seasonal amplitudes of PDO, MEI, and NPGO provided a moderate fit for Pacific cod CPUE (deviance explained$=0.581$). Lagged NPGO was also a good predictor of rougheye rockfish CPUE and provided a good fit to the data (deviance explained$=0.641$).

## Discussion

Deepwater species such as the groundfish examined in this study are difficult to monitor regularly and study extensively, so determining environmental variables that most accurately predict population health of these species is critical. In this study, I illustrated LASSO-type variable selection on the SIVCM capable of including spatiotemporal covariates for modeling large-scale population data, identifying the most significant factors affecting catch and mean weights of groundfish, and determining the functional relationships of these important predictors to the response for inference. Though the environmental variables considered in this application were collected in situ, the SIVCM with group LASSO-type selection would be ideal for spatially or spatiotemporally measured remotely sensed data, such as environmental satellite data.

CPUE and mean weights of groundfish contribute to calculations of relative population numbers and weights which are used to inform management and policy decisions^85^. The use of relevant environmental covariates influencing groundfish catch and weight can inform spatiotemporal modeling of groundfish distributions and aid prediction of distribution shifts due to climate change^86^. This knowledge is particularly urgent for predicting the effects of climate change and devising adaptive strategies for vulnerable populations such as the three rockfish species included in this study, stocks of which are highly sensitive to climate change in the eastern Bering Sea^87^.

Increased growth rates in juvenile sablefish with warmer water temperatures have been observed in laboratory settings^88,89^ and off the coast of Oregon, where early growth was related to recruitment^90^. Increased sablefish recruitment may be reliant on greater stratification developed by warmer surface water temperatures^91^, while warmer deep waters during the sablefish egg stage combined with colder surface waters during the pelagic larvae stage have been related to higher recruitment^92^. The importance of sea surface temperature to sablefish CPUE across all years established in this analysis is therefore in agreement with previous experimental and observational studies and stresses the importance of surface water temperatures on the success of juvenile sablefish^90^. Sogard^90^ presumed faster sablefish growth occurred due to high overall productivity reflected in the PDO index, however in this analysis there was no direct relationship between PDO and sablefish CPUE or mean weight, nor was PDO found to be related to to the selection of any environmental variables for sablefish CPUE or weight. However, the selection of silicate as important for sablefish CPUE may point to a relationship of primary production on sablefish, as silicate is an important regulator of phytoplankton growth in Alaskan marine waters^93^. The selection of air temperature and wind speed as important across all years for sablefish weight is consistent with the relationships of transport and of July winds during juvenile years to recruitment^92,94^ and suggests the importance of air and surface water movements on early-stage sablefish.

Chlorophyll and sea surface temperature were important across all years for Pacific halibut CPUE, along with air temperature, dissolved oxygen, and wave height. These findings are corroborated by previous observations of Pacific halibut. Temperature influences growth on Pacific halibut juveniles, which exhibit compensatory growth in response to periods of temperature-limited growth^95,96^. Bioenergetics model simulations and diet data suggest that adult growth may also be limited by temperature and prey quality or availability^97^. Pacific halibut weight data were no longer collected after 1994 by the AFSC surveys, so a consistent, long term signal in the data to determine important variables related to weight of Pacific halibut was unlikely. However, growth in early years contributes to year-class abundance for Pacific halibut^95^. The findings of chlorophyll, air temperature, and wave height as important to Pacific halibut CPUE point to sensitivity of Pacific halibut to bottom-up forces in the North Pacific Ocean^98^. Chlorophyll is an important indicator of changes in the carbon transport system and plankton biomass vital for energy transfer to higher tropic levels^69,99^, while air temperature and wave height influence the duration of wind-mixing events and change the depth of the mixed-layer, affecting primary and secondary production^7,100–102^. A relationship between dissolved oxygen and Pacific halibut catch has also been established previously, with Pacific halibut likely adjusting to changes in oxygen environments through lateral rather than vertical movements^103^.

Variables selected as important to specific groundfish in this work that have not previously been explored encourage further investigation. For example, only deep water alkalinity contributed to Pacific cod CPUE across all years of available data, but Pacific cod CPUE was also related to PDO, MEI, and NPGO indices. While water temperatures have been related to interannual depth changes, range shifts, and population declines of adult Pacific cod^104–106^, juvenile Pacific cod may respond to drivers other than temperature^106^. Alkalinity, as part of the ocean’s carbonate system, moderates seasonal pH variability^107^. Further, increased variability in the interannual carbonate system in Pacific Ocean is also attributed to variability in the PDO and ENSO^108^. Therefore the selection of alkalinity as important to Pacific cod catch may correspond to several climate modes strongly dictated by the carbonate system and closely correlated to variability in alkalinity. Then again, the importance of alkalinity may instead represent a sensitivity of early stage Pacific cod to ocean acidification, as increased atmospheric carbon dioxide changes alkalinity levels^109,110^.

Another example of previously unexplored environmental variables found in this study to be related to groundfish are the important variables for rougheye rockfish CPUE across all years, which were exclusively from deeper waters (>300 m). Since rockfish are viviparous, exposure to near-surface environment during development is eliminated. Rougheye rockfish CPUE was best predicted by NPGO seasonal amplitudes, while chlorophyll, dissolved oxygen, and silicate were all important for rougheye rockfish CPUE over all years. NPGO describes variations in salinity, nitrate, phosphate, silicate, oxygen, and chlorophyll, capturing important interannual and decadal biological patterns^4^. This suggests that the selection of variables affecting rougheye rockfish catch was the result of an existing, strong effect of nutrient cycling patterns captured by the NPGO index on rougheye rockfish populations.

Selection of important variables related to groundfish catch and weight using LSSGLASSO for SIVCMs provided important confirmation of previous findings and new avenues of research for large populations of groundfish in the North Pacific over an extended time period during which several large-scale regime shifts have been reported^1,6,111,112^. While large-scale climate indices are often favored when studying populations affected by regime shifts of the northern Pacific marine system, the specific pathways of their effects on a population or community are often difficult to disentangle. Most groundfish responses had a large array of environmental variables selected for specific years, indicating that the strength of environmental effects on groundfish may vary over time. Selection of environmental variables appeared to be unrelated to seasonal amplitudes of the PDO, MEI, and NPGO climate indices for most groundfish. Large-scale climate patterns are associated with groundfish responses^1,2^, however my results highlight the insufficiency of climate modes alone to accurately describe variability found in many commercially valuable marine populations on decadal scales^10^. These results indicate the existence of several pathways of climate and other environmental effects on groundfish responses. Ecological variability in these marine systems are therefore likely driven by gradual spatial and temporal climate variability that reduces resilience, with abrupt changes in climate modes precipitating permanent changes in vulnerable systems^8^.

## References

[1] Francis RC, Hare SR, Hollowed AB, Wooster WS (1998). Effects of interdecadal climate variability on the oceanic ecosystems of the NE Pacific. Fish. Oceanogr..

[2] Hollowed AB, Wooster WS (1992). Variability of winter ocean conditions and strong year classes of Northeast Pacific groundfish. ICES Mar. Sci. Symp..

[3] Mantua NJ, Hare SR (2002). The Pacific decadal oscillation. J. Oceanogr..

[4] Di Lorenzo E (2008). North Pacific gyre oscillation links ocean climate and ecosystem change. Geophys. Res. Lett..

[5] Di Lorenzo E (2013). Synthesis of pacific ocean climate and ecosystem dynamics. Oceanography.

[6] Anderson PJ, Piatt JF (1999). Community reorganization in the Gulf of Alaska following ocean climate regime shift. Mar. Ecol. Prog. Ser..

[7] Polovina, J. J., Mitchum, G. T. & Evans, G. T. Decadal and basin-scale variation in mixed layer depth and the impact on biological production in the Central and North Pacific, 1960–88. *Deep Sea Res. Part I Oceanogr. Res. Pap.* **42**, 1701–1716 (1995).

[8] Litzow MA, Mueter FJ (2014). Assessing the ecological importance of climate regime shifts: an approach from the North Pacific Ocean. Prog. Oceanogr..

[9] Möllmann, C., Folke, C., Edwards, M. & Conversi, A. Marine regime shifts around the globe: theory, drivers and impacts. *Philos. Trans. R. Soc. B Biol. Sci.* **370**, 20130260. 10.1098/rstb.2013.0260 (2015).

[10] Litzow MA, Mueter FJ, Hobday AJ (2014). Reassessing regime shifts in the North Pacific: incremental climate change and commercial fishing are necessary for explaining decadal-scale biological variability. Glob. Change Biol..

[11] Goen, J., & Erikson, L. *Fishery Statistics*. Technical Report. IPHC-2018-AM094-05, International Pacific Halibut Commission (2017).

[12] Johnson, K. F. *et al.**Status of the U.S. Sablefish Resource in 2015*. Technical Report. Pacific Fishery Management Council (2016).

[13] Pacific Fishery Management Council. *Pacific Coast Groundfish Fishery Management Plan*. Technical Report, NOAA (2016).

[14] NPFMC. *Fishery Management Plan for Groundfish of the Gulf of Alaska*. Technical Report, North Pacific Fishery Management Council (2017).

[15] Pennoyer, S. & Balsiger, J. *Groundfish Total Allowable Catch Specifications and Prohibited Species Catch Limits Under the Authority of the Fishery Management Plans for the Groundfish Fishery of the Bering Sea and Aleutian Islands Area and Groundfish of the Gulf of Alaska: Final Supplemental Environmental Impact Statement*. Technical Report, United States National Marine Fisheries Service Alaska Regional Office, Juneau, Alaska (1998).

[16] Rodgveller CJ, Lunsford CR, Fujioka JT (2008). Evidence of hook competition in longline surveys. Fish. Bull..

[17] Hutchings JA (2000). Collapse and recovery of marine fishes. Nature.

[18] Moore JA (1999). Deep-sea finfish fisheries: lessons from history. Fisheries.

[19] Moore J, Mace P (1999). Challenges and prospects for deep-sea finfish fisheries. Fisheries.

[20] Rijnsdorp AD, Peck MA, Engelhard GH, Möllmann C, Pinnegar JK (2009). Resolving the effect of climate change on fish populations. ICES J. Mar. Sci. J. Conseil.

[21] Xia Y, Li WK (1999). On single-index coefficient regression models. J. Am. Stat. Assoc..

[22] Kammann EE, Wand MP (2003). Geoadditive models. J. R. Stat. Soc. Ser. C (Appl. Stat.).

[23] Lu, Z., Steinskog, D. J., Tjøstheim, D. & Yao, Q. Adaptively varying-coefficient spatiotemporal models. *J. R. Stat. Soc. Ser. B (Stat. Methodol.)* **71**, 859–880. 10.1111/j.1467-9868.2009.00710.x (2009).

[24] Ruppert, D., Wand, M. P. & Carroll, R. J. *Semiparametric Regression. Cambridge Series in Statistical and Probabilistic Mathematics* (Cambridge University Press, 2003).

[25] Scheipl F, Staicu A-M, Greven S (2015). Functional additive mixed models. J. Comput. Graph. Stat..

[26] Wood, S. N. *Generalized Additive Models. Texts in Statistical Science Series* (Chapman & Hall/CRC, 2006). An Introduction with $$R$$.

[27] Wood SN, Scheipl F, Faraway JJ (2013). Straightforward intermediate rank tensor product smoothing in mixed models. Stat. Comput..

[28] Conn PB, Johnson DS, Boveng PL (2015). On extrapolating past the range of observed data when making statistical predictions in ecology. PLoS ONE.

[29] Hodges JS, Reich BJ (2010). Adding spatially-correlated errors can mess up the fixed effect you love. Am. Stat..

[30] Kim M, Wang L (2020). Generalized spatially varying coefficient models. J. Comput. Graph. Stat..

[31] Mu J, Wang G, Wang L (2018). Estimation and inference in spatially varying coefficient models. Environmetrics.

[32] Brumback BA, Rice JA (1998). Smoothing spline models for the analysis of nested and crossed samples of curves. J. Am. Stat. Assoc..

[33] Augustin NH, Trenkel VM, Wood SN, Lorance P (2013). Space-time modelling of blue ling for fisheries stock management. Environmetrics.

[34] Finley AO (2011). Comparing spatially-varying coefficients models for analysis of ecological data with non-stationary and anisotropic residual dependence. Methods Ecol. Evol..

[35] Al-Sulami D, Jiang Z, Lu Z, Zhu J (2017). Estimation for semiparametric nonlinear regression of irregularly located spatial time-series data. Econom. Stat..

[36] Gelfand AE, Kim H-J, Sirmans CF, Banerjee S (2003). Spatial modeling with spatially varying coefficient processes. J. Am. Stat. Assoc..

[37] Feng S, Xue L (2015). Model detection and estimation for single-index varying coefficient model. J. Multivariate Anal..

[38] Zhao P, Xue L (2009). Variable selection for semiparametric varying coefficient partially linear models. Stat. Probab. Lett..

[39] Guisan A, Hastie T (2002). Generalized linear and generalized additive models in studies of species distributions: setting the scene. Ecol. Model..

[40] Zhang L, Gove JH (2005). Spatial assessment of model errors from four regression techniques. For. Sci..

[41] Cai A, Tsay RS, Chen R (2009). Variable selection in linear regression with many predictors. J. Comput. Graph. Stat..

[42] Tibshirani, R. Regression shrinkage and selection via the lasso. *J. R. Stat. Soc. Ser. B (Stat. Methodol.)* **58**, 267–288. 10.1111/j.2517-6161.1996.tb02080.x (1996).

[43] Ledolter, J. *Penalty-Based Variable Selection in Regression Models with Many Parameters (LASSO)*, chap. 6, 71–82 (Wiley, 2013). https://onlinelibrary.wiley.com/doi/pdf/10.1002/9781118596289.ch6.

[44] Yuan, M. & Lin, Y. Model selection and estimation in regression with grouped variables. *J. R. Stat. Soc. Ser. B (Stat. Methodol.)* **68**, 49–67. 10.1111/j.1467-9868.2005.00532.x (2006).

[45] Fan, J., Yao, Q. & Cai, Z. Adaptive varying-coefficient linear models. *J. R. Stat. Soc. Ser. B (Stat. Methodol.)* **65**, 57–80 (2003).

[46] Matsui H, Misumi T (2015). Variable selection for varying-coefficient models with the sparse regularization. Comput. Stat..

[47] Wang H, Xia Y (2009). Shrinkage estimation of the varying coefficient model. J. Am. Stat. Assoc..

[48] Xue L, Qu A (2012). Variable selection in high-dimensional varying-coefficient models with global optimality. J. Mach. Learn. Res..

[49] Feng S, Xue L (2013). Variable selection for single-index varying-coefficient model. Front. Math. China.

[50] Song Y, Jian L, Lin L (2016). Robust exponential squared loss-based variable selection for high-dimensional single-index varying-coefficient model. J. Comput. Appl. Math..

[51] Yang J, Yang H (2017). Robust modal estimation and variable selection for single-index varying-coefficient models. Commun. Stat. Simul. Comput.

[52] Wei F, Huang J, Li H (2011). Variable selection and estimation in high-dimensional varying-coefficient models. Stat. Sin..

[53] Sun W, Bindele HF, Abebe A, Correia HE (2021). Robust functional coefficient selection for the single-index varying coefficients regression model. J. Stat. Comput. Simul..

[54] Alaska Fisheries Science Center. *AFSC/ABL: Longline Sablefish Survey*. https://noaa-fisheries-afsc.data.socrata.com/dataset/AFSC-ABL-Longline-Sablefish-Survey/itxd-qjvg/data (2019). Accessed 14 Apr 2014.

[55] Sigler, M. F. & Lunsford, C. R. *Survey Protocol for the Alaska Sablefish Longline Survey*. Technical Report, Alaska Fisheries Science Center (2009).

[56] National Data Buoy Center. Meteorological and oceanographic data collected from the National Data Buoy Center Coastal-Marine Automated Network (C-MAN) and moored (weather) buoys. https://accession.nodc.noaa.gov/NDBC-CMANWx (2018).

[57] Alaska Fisheries Science Center. AFSC/RACE/GAP: RACEBASE Database. Online: http://www.afsc.noaa.gov/RACE/groundfish/survey_data/default.htm (2019).

[58] O’Brien, T. D. *COPEPOD: The Global Plankton Database. A Review of the 2007 Database Contents and New Quality Control Methodology*. Technical Report. NOAA Tech. Memo. NMFS-F/ST-34, U.S. Dep. Commerce (2007).

[59] Boyer, T. P. *et al.**World Ocean Database 2013*. Technical Report. National Oceanographic Data Center, Ocean Climate Laboratory, NOAA (2013). 10.7289/V5NZ85MT.

[60] Neter, J., Kutner, M. H., Nachtsheim, C. J. & Wasserman, W. *Applied Linear Statistical Models*, vol. 4 (Irwin Chicago, 1996).

[61] O’Brien RM (2007). A caution regarding rules of thumb for variance inflation factors. Qual. Quant..

[62] Li L, Losser T, Yorke C, Piltner R (2014). Fast inverse distance weighting-based spatiotemporal interpolation: a web-based application of interpolating daily fine particulate matter pm2.5 in the contiguous U.S. using parallel programming and k–d tree. Int. J. Environ. Res. Public Health.

[63] Melo, C. & Melo, O. *geosptdb: Spatio-Temporal Inverse Distance Weighting and Radial Basis Functions with Distance-Based Regression* (2015). R package version 0.5-0.

[64] Whitney FA (2011). Nutrient variability in the mixed layer of the subarctic Pacific Ocean, 1987–2010. J. Oceanogr..

[65] Whitney FA, Bograd SJ, Ono T (2013). Nutrient enrichment of the subarctic Pacific Ocean pycnocline. Geophys. Res. Lett..

[66] Brodeur RD, Ware DM (1992). Long-term variability in zooplankton biomass in the subarctic Pacific Ocean. Fish. Oceanogr..

[67] Chiba S, Tadokoro K, Sugisaki H, Saino T (2006). Effects of decadal climate change on zooplankton over the last 50 years in the western subarctic North Pacific. Glob. Change Biol..

[68] Childers, A. R., Whitledge, T. E. & Stockwell, D. A. Seasonal and interannual variability in the distribution of nutrients and chlorophyll a across the Gulf of Alaska shelf: 1998–2000. *Deep Sea Res. Part II Top. Stud. Oceanogr.* **52**, 193–216. 10.1016/j.dsr2.2004.09.018 (2005). U.S. GLOBEC Biological and Physical Studies of Plankton, Fish and Higher Trophic Level Production, Distribution, and Variability in the Northeast Pacific.

[69] Sackmann B, Mack L, Logsdon M, Perry MJ (2004). Seasonal and inter-annual variability of SeaWiFS-derived chlorophyll a concentrations in waters off the Washington and Vancouver Island coasts, 1998–2002. Deep Sea Res. Part II Top. Stud. Oceanogr..

[70] Wong C (2002). Seasonal cycles of nutrients and dissolved inorganic carbon at high and mid latitudes in the North Pacific Ocean during the Skaugran cruises: determination of new production and nutrient uptake ratios. Deep Sea Res. Part II Top. Stud. Oceanogr..

[71] Houde, E. D. chap. Recruitment variability. In *Fish Reproductive Biology: Implications for Assessment and Management* (Eds. Jakobsen, T., Fogarty, M. J., Megrey, B. A. & Moksness, E.) (Wiley, 2016).

[72] Mason JC, Beamish RJ, McFarlane GA (1983). Sexual maturity, fecundity, spawning, and early life history of sablefish (Anoplopoma fimbria) off the Pacific Coast of Canada. Can. J. Fish. Aquat. Sci..

[73] Stark JW (2007). Geographic and seasonal variations in maturation and growth of female Pacific cod (Gadus macrocephalus) in the Gulf of Alaska and Bering Sea. Fish. Bull..

[74] Clark WG, Hare SR, Parma AM, Sullivan PJ, Trumble RJ (1999). Decadal changes in growth and recruitment of Pacific halibut (*Hippoglossus stenolepis*). Can. J. Fish. Aquat. Sci..

[75] Echeverria TW (1987). Thirty-four species of California rockfishes: maturity and seasonality of reproduction. Fish. Bull..

[76] Di Lorenzo, E. North Pacific Gyre Oscillation (2018). NPGO index.

[77] NOAA ESRL Physical Sciences Division. Multivariate ENSO Index Version 2 (MEI.v2) (2019). ENSO index.

[78] Mantua, N. J. & JISAU, University of Washington. The Pacific Decadal Oscillation. http://research.jisao.washington.edu/pdo/ (2016).

[79] Di Lorenzo E (2010). Central Pacific El Niño and decadal climate change in the North Pacific Ocean. Nat. Geosc..

[80] Ladd, C. & Stabeno, P. J. Stratification on the Eastern Bering Sea shelf revisited. *Deep Sea Res. Part II Top. Stud. Oceanogr.* **65-70**, 72–83. 10.1016/j.dsr2.2012.02.009 (2012). Understanding Ecosystem Processes in the Eastern Bering Sea.

[81] Benjamini, Y. & Hochberg, Y. Controlling the false discovery rate: a practical and powerful approach to multiple testing. *J. R. Stat. Soc. Ser. B (Methodol.)* **57**, 289–300 (1995).

[82] Frainer, A. *et al.* Climate-driven changes in functional biogeography of Arctic marine fish communities. *Proc. Natl. Acad. Sci.* **114**, 12202–12207, 10.1073/pnas.1706080114 (2017). 10.1073/pnas.1706080114 PMC5699037 29087943

[83] Hewitt JE, Ellis JI, Thrush SF (2016). Multiple stressors, nonlinear effects and the implications of climate change impacts on marine coastal ecosystems. Glob. Change Biol..

[84] Liu H (2012). Nonlinear dynamic features and co-predictability of the Georges Bank fish community. Mar. Ecol. Prog. Ser..

[85] Echave, K., Rodgveller, C. & Shotwell, S. K. *Calculation of the Geographic Area Sizes Used to Create Population Indices for the Alaska Fisheries Science Center Longline Survey*. Technical Report. NOAA Tech. Memo. NMFS-AFSC-253, U.S. Department of Commerce (2013).

[86] Webster RA, Soderlund E, Dykstra CL, Stewart IJ (2020). Monitoring change in a dynamic environment: spatio-temporal modelling of calibrated data from different types of fisheries surveys of Pacific halibut. Can. J. Fish. Aquat. Sci..

[87] Spencer PD, Hollowed AB, Sigler MF, Hermann AJ, Nelson MW (2019). Trait-based climate vulnerability assessments in data-rich systems: an application to eastern Bering Sea fish and invertebrate stocks. Glob. Change Biol..

[88] Sogard SM, Olla BL (2001). Growth and behavioral responses to elevated temperatures by juvenile sablefish *Anoplopoma fimbria* and the interactive role of food availability. Mar. Ecol. Prog. Ser..

[89] Stoner AW, Sturm EA (2004). Temperature and hunger mediate sablefish (*Anoplopoma fimbria*) feeding motivation: implications for stock assessment. Can. J. Fish. Aquat. Sci..

[90] Sogard S (2011). Interannual variability in growth rates of early juvenile sablefish and the role of environmental factors. Bull. Mar. Sci..

[91] Shotwell, S. K., Hanselman, D. H. & Belkin, I. M. Toward biophysical synergy: investigating advection along the Polar Front to identify factors influencing Alaska sablefish recruitment. *Deep Sea Res. Part II Top. Stud. Oceanogr.* **107**, 40–53. 10.1016/j.dsr2.2012.08.024 (2014). Fronts, Fish and Top Predators.

[92] Tolimieri N, Haltuch MA, Lee Q, Jacox MG, Bograd SJ (2018). Oceanographic drivers of sablefish recruitment in the California current. Fish. Oceanogr..

[93] Harrison PJ, Whitney FA, Tsuda A, Saito H, Tadokoro K (2004). Nutrient and plankton dynamics in the NE and NW gyres of the subarctic Pacific Ocean. J. Oceanogr..

[94] Coffin, B. & Mueter, F. Environmental covariates of sablefish (Anoplopoma fimbria) and Pacific ocean perch (Sebastes alutus) recruitment in the Gulf of Alaska. *Deep Sea Res. Part II Top. Stud. Oceanogr.* **132**, 194–209. 10.1016/j.dsr2.2015.02.016 (2016). Understanding Ecosystem Processes in the Gulf of Alaska: Volume 1.

[95] Hagen, P. T. & Quinn, T. J. Long-term growth dynamics of young Pacific halibut: evidence of temperature-induced variation. *Fish. Res.* **11**, 283–306. 10.1016/0165-7836(91)90006-2 (1991). Fish Population Dynamics: Solving Fishery Management Problems.

[96] Hurst TP, Spencer ML, Sogard SM, Stoner AW (2005). Compensatory growth, energy storage and behavior of juvenile Pacific halibut *Hippoglossus stenolepis* following thermally induced growth reduction. Mar. Ecol. Prog. Ser..

[97] Holsman KK, Aydin K, Sullivan J, Hurst T, Kruse GH (2019). Climate effects and bottom-up controls on growth and size-at-age of Pacific halibut (*Hippoglossus stenolepis*) in Alaska (USA). Fish. Oceanogr..

[98] Lynam CP (2017). Interaction between top-down and bottom-up control in marine food webs. Proc. Natl. Acad. Sci..

[99] Desmit X, Ruddick K, Lacroix G (2015). Salinity predicts the distribution of chlorophyll a spring peak in the southern North Sea continental waters. J. Sea Res..

[100] Benson AJ, Trites AW (2002). Ecological effects of regime shifts in the Bering Sea and eastern North Pacific Ocean. Fish Fish..

[101] Feng J (2015). Contrasting correlation patterns between environmental factors and chlorophyll levels in the global ocean. Glob. Biogeochem. Cycles.

[102] Kahru M (2010). Global correlations between winds and ocean chlorophyll. J. Geophys. Res. Oceans.

[103] Sadorus LL, Mantua NJ, Essington T, Hickey B, Hare S (2014). Distribution patterns of Pacific halibut (*Hippoglossus stenolepis*) in relation to environmental variables along the continental shelf waters of the US West Coast and southern British Columbia. Fish. Oceanogr..

[104] Barbeaux, S. *et al.**Gulf of Alaska Stock Assessments*. Technical Report, North Pacific Fishery Management Council, Anchorage, AK (2018).

[105] Barbeaux SJ, Hollowed AB (2018). Ontogeny matters: climate variability and effects on fish distribution in the eastern Bering Sea. Fish. Oceanogr..

[106] Yang, Q. *et al.* How “The Blob” affected groundfish distributions in the Gulf of Alaska. *Fish. Oceanogr.* **28**, 434–453. 10.1111/fog.12422 (2019).

[107] Hagens M, Middelburg JJ (2016). Attributing seasonal pH variability in surface ocean waters to governing factors. Geophys. Res. Lett..

[108] Fry CH, Tyrrell T, Hain MP, Bates NR, Achterberg EP (2015). Analysis of global surface ocean alkalinity to determine controlling processes. Mar. Chem..

[109] Bromhead, D. *et al.* The potential impact of ocean acidification upon eggs and larvae of yellowfin tuna (*Thunnus albacares*). *Deep Sea Res. Part II Top. Stud. Oceanogr.* **113**, 268–279, 10.1016/j.dsr2.2014.03.019 (2015). Impacts of climate on marine top predators.

[110] Doney, S. C. *et al.* Impact of anthropogenic atmospheric nitrogen and sulfur deposition on ocean acidification and the inorganic carbon system. *Proc. Natl. Acad. Sci.* **104**, 14580–14585. 10.1073/pnas.0702218104 (2007). 10.1073/pnas.0702218104 PMC1965482 17804807

[111] Napp JM, Hunt GL (2001). Anomalous conditions in the south-eastern Bering Sea 1997: linkages among climate, weather, ocean, and biology. Fish. Oceanogr..

[112] Noakes DJ, Beamish RJ (2009). Synchrony of marine fish catches and climate and ocean regime shifts in the North Pacific Ocean. Mar. Coast. Fish..

